# Supplementary material for: Convoluted micellar morphological transitions driven by tailorable mesogenic ordering effect from discotic mesogen-containing block copolymer
Source: Nat Commun. 2024 Apr 6;15:2968. doi: 10.1038/s41467-024-47312-6 (PMC10997646; doi:10.1038/s41467-024-47312-6)
Supplement: Supplementary file 1 — Supplementary Information [file 41467_2024_47312_MOESM1_ESM.pdf]

Supplementary Information for

Convoluted Micellar Morphological Transitions Driven by Tailorable Mesogenic Ordering Effect from Discotic Mesogen-Containing Block Copolymer

Huanzhi Yang[1], Yunjun Luo[1, 2], Bixin Jin[*1], Shumeng Chi[1, 3], Xiaoyu Li[*1, 2, 3]

[1.] School of Materials Science and Engineering. Beijing Institute of Technology, Beijing 100081, China.

[2.] Key Laboratory of High Energy Density Materials, MOE. Beijing Institute of Technology, Beijing 100081, China.

[3.] Experimental Center of Advanced Materials, Beijing Institute of Technology, Beijing 100081, China.

E-mail: bixinjin@bit.edu.cn; xiaoyuli@bit.edu.cn;

Supplementary Methods

Nuclear magnetic resonance (NMR). NMR spectra were recorded using an Avance 500 (Bruker A.G.) instrument (operating at 400 MHz) at room temperature (r.t., 21 °C).

Matrix-assisted laser desorption/ionization time of flight mass spectrometry (MALDI-TOF-MS). Mass spectra were performed on a MALDI-TOF-MS (FlashDetector, Bruker ultraflextreme).

Fourier transform infrared spectroscopy (FT-IR). FT-IR spectra were obtained on a Thermo Fisher Scientific Nicolet iS50 FT-IR instrument equipped with an ATR unit at r.t.

Element analysis (EA). EA was performed with a Vario EL cube instrument (Elementar, Germany).

Differential scanning calorimetry (DSC) and thermogravimetric analysis (TGA). DSC and TGA were performed on the Q100, and Q500 from TA instruments, respectively, under nitrogen at a heating/cooling rate of 10 °C/min. Typically, about 5 mg of the solid sample was encapsulated in a sealed aluminum pan with an identical empty pan as the reference.

The above methods apply to homopolymers. To observe the LC to the amorphous phase transition of P*t*BA-*b*-PHATMA diblock copolymer, we had to anneal the sample at 35 °C for 24 h before the DSC characterization and record the results from the first heating scan.

UV-Visible and fluorescence spectrometer. UV-vis data were acquired from a Lambda 35 spectrometer using standard quartz cells from the wavelength of 200 nm to 700 nm. Fluorescence data were obtained from a PerkinElmer LS 45 fluorescence spectrometer.

Grazing-incident wide-angle X-ray scattering (GI-WAXS). GI-WAXS measurements were performed at beamline BL16B1, the microfocus beamline at Shanghai Synchrotron Radiation Facility (SSRF).[1] The X-ray energy was 10 keV, corresponding to the wavelength of 1.24 Å. Diffraction patterns were collected on a PILATUS3 X 2 M detector, the pixel size was 172 μm×172 μm, and the maximum frame rate was 250 Hz. A fixed X-ray incident angle of 0.15° was used, which gave a

footprint on the sample surface of ~500×2 μm^2. The results were obtained by using the software SGTools.[2] Out-of-plane line profiles were extracted by integration of a sector of data along q_z with an angular width of 25°. In-plane lines were extracted by integration of a box of data along q_{xy}. These integration regions are shown in Supplementary Figure 28.

Polarizing optical microscopy (POM). POM was carried out on a Leica DM2500P microscope equipped with a Linkam THMS600 hot stage. The samples (0.5 mg) were prepared by melt-pressing and sandwiched between two pieces of glass. The heating and cooling rates in the experiments were 10 °C/min.

Synthesis of 1,2-Di(hexyloxy)benzene

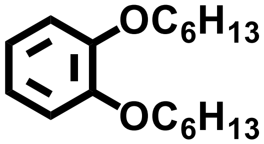

In a three-neck flask under nitrogen atmosphere, catechol (44.0 g, 399.6 mmol), 1-bromohexane (263.8 g, 1598.4 mmol), K_2CO_3 (220.9 g, 1598.4 mmol), KI (catalytic quantity) were stirred in a mixture of acetone and EtOH (1/1, volume ratio) (250 mL) and heated at reflux for 24 h. The organic layer was filtered and the solvent was removed under a vacuum. The crude product was purified *via* column chromatography (silica gel, PE/CH_2Cl_2 = 10:1, volume ratio). The pale-yellow oil was obtained with a yield of 95% (105.70 g). ^{1}H NMR δ_H (ppm) (400 MHz, $CDCl_3$): 6.89 (s, 4H), 4.01-3.98 (t, 4H), 1.84-1.79 (m, 4H), 1.51-1.45 (m, 4H), 1.36-1.33 (m, 8H), 0.92-0.89 (t, 6H).

Synthesis of 2,3,6,7,10,11-Hexakis(hexyloxy)triphenylene

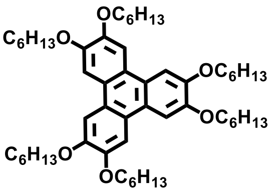

In a three-neck flask under a nitrogen atmosphere, 1,2-di(hexyloxy)benzene (20.0 g, 71.8 mmol) was added to a vigorously-stirred suspension of iron (III) trichloride (34.95 g, 215.5 mmol) in anhydrous CH_2Cl_2 (80 mL). The mixture was stirred at r.t. for 3 h with 3 mL H_2SO_4 as the catalyst. The mixture was added to MeOH (500 mL) to quench the reaction. The mixture was filtered and purified *via* column chromatography (silica gel, PE/CH_2Cl_2 = 4:1, volume ratio) to afford a white solid (14.89 g, 75%). ^{1}H NMR δ_H (ppm) (400 MHz, $CDCl_3$): 7.84 (s, 6H), 4.25-4.22 (t, 12H), 1.98-1.91 (m, 12H), 1.62-1.55 (m, 12H), 1.45-1.36 (m, 24H), 0.96-0.92 (s, 18H).

Synthesis of 2-Hydroxy-3, 6, 7, 10, 11-penta(hexyloxy)triphenylene

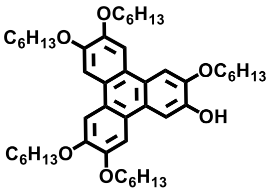

BBr_3 (28.6 g, 110 mmol) was added dropwise to a vigorously stirred suspension of catechol (11 g, 110 mmol) in anhydrous CH_2Cl_2 (70 mL) until the initial suspension became yellow solution at 0 ℃. The reaction was continued for 3 h at r.t. The solvent was removed and the product was distilled under a vacuum to give B-bromocatecholboronane as a white solid (15 g, 75%). The solid was then used to make a 0.5 M solution by mixing with CH_2Cl_2 (150 mL) and this was used for the next ether cleavage reactions.

A solution of 2,3,6,7,10,11-hexakis(hexyloxy)triphenylene (16.43 g, 20 mmol) was dissolved in anhydrous CH_2Cl_2 (140 mL) and cooled to 0 °C. The B-bromocatecholboronane solution in CH_2Cl_2 (48 mL, 24 mmol) was added under a

nitrogen atmosphere and the mixture was stirred at r.t. for 24 h. The mixture was poured over ice water and extracted with CH_2Cl_2. The combined extract was dried with anhydrous Na_2SO_4 overnight. The solvent was removed under a vacuum and the crude product was purified *via* column chromatography (silica gel, PE/CH_2Cl_2 = 4:3, volume ratio) to afford a white solid (5.87 g, 40%), which was recrystallized in EtOH. ^{1}H NMR δ_H (ppm) (400 MHz, $CDCl_3$): 7.96 (s, 1H), 7.83-7.77 (m, 5H), 5.90 (s, 1H), 4.30-4.19 (m, 10H), 1.97-1.91 (m, 10H), 1.61-1.55 (m, 10H), 1.43-1.40 (m, 20H), 0.95-0.92(m, 15H).

Synthesis of 2-(6-Hydroxylhexyloxy)-3,6,7,10,11-pentakis(hexyloxy)triphenylene

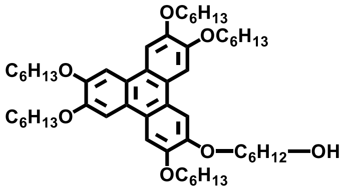

In a three-neck flask under nitrogen atmosphere, 2-hydroxyl-3,6,7,10,11-pentakis(hexyloxy)triphenylene (2.06 g, 2.8 mmol), 6-bromo-1-hexanol (1.0 g, 5.6 mmol), K_2CO_3 (0.387 g, 5.6 mmol) and KI (catalytic quantity) were stirred in CH_3CN (60 mL) and heated at 80 °C for 24 h. After cooling to r.t., the organic layer was filtered and the solvent was removed under reduced pressure. The crude product was purified *via* column chromatography (silica gel, PE/CH_2Cl_2 = 1:4, volume ratio). A white solid was obtained with a yield of 85% (1.90 g). ^{1}H NMR δ_H (ppm) (400 MHz, $CDCl_3$): 7.84 (s, 6H), 4.25-4.22 (t, 12H), 3.73-3.67 (m, 2H), 1.98-1.90 (m, 12H), 1.67-1.51 (m, 12H), 1.43-1.36 (m, 24H), 0.95-0.92 (t, 15H).

Synthesis of 6-[3,6,7,10,11-Pentakis(hexyloxy)-2-oxytriphenylene]hexyl methacrylate

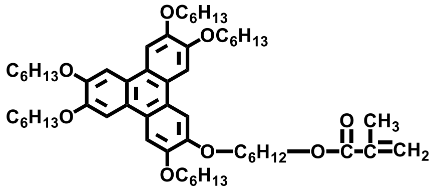

In a three-neck flask under nitrogen atmosphere, 2-(6-hydroxylhexyloxy)-3,6,7,10,11-pentakis(hexyloxy)triphenylene (0.9 g, 1.06 mmol) and TEA (0.33 mL, 2.34 mmol) were dissolved in dry CH_2Cl_2 (20 mL) and cooled to 0 °C. Methacryoyl chloride (0.2 mL, 2.12 mmol) was added dropwise to the reaction mixture. The solution was allowed to warm up to r.t. and stirred for 10 h. After evaporation of the solvent, the crude product was purified *via* column chromatography (silica gel, PE/CH_2Cl_2 = 4:1, volume ratio). A white solid was obtained with a yield of 45% (0.86 g). ^{1}H NMR δ_H (ppm) (400 MHz, $CDCl_3$): 7.84 (s, 6H), 6.10 (s, 1H), 5.54-5.53 (t, 1H), 4.25-4.17 (m, 14H), 1.98-1.90 (m, 15H), 1.68-1.52 (m, 12H), 1.43-1.36 (m, 24H), 0.95-0.92 (m, 15H); ^{13}C NMR δ_H (ppm) (101 MHz, $CDCl_3$): 167.67, 149.16, 149.02, 136.65, 125.35, 123.84, 123.79, 123.73, 107.50, 69.86, 69.78, 69.67, 64.80, 31.84, 29.58, 28.80, 26.04, 26.01, 22.81, 18.48, 12.20; FT-IR (cm^{-1}): 836.79, 1049.43, 1168.74, 1257.72, 1386.26, 1435.03, 1515.98, 1616.60, 1719.62, 2856.10, 2925.04; MALDI-TOF-MS: calcd. for

$C_{58}H_{88}O_8$ 913.3, found 912.8; EA: calcd. for $C_{58}H_{88}O_8$ C, 76.27; H, 9.71; O, 14.01; found: C, 74.47; H, 9.57; O, 15.95.

Synthesis of 2,4,7-Trinitro-9H-fluoren-9-one (TNF)

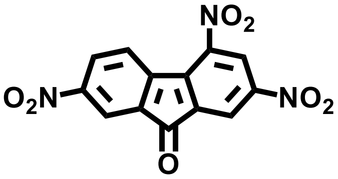

Into a stirred solution of HNO_3 (15.8 M, 40 mL) and H_2SO_4 (18 M, 40 mL), 9-fluorenone (1.80 g, 0.01 mol) was added at r.t. and heated to reflux for 2 h (~105 °C, internal). The solution was allowed to warm up to r.t. and the reaction mixture was then poured into 200 mL ice water. The precipitated powder was collected by suction filtration, washed with water, and dried. The yellow solid was further purified *via* repeated precipitations from CH_2Cl_2 solution into MeOH, and dried under reduced pressure. A yellowish solid was obtained as the final product (2.6 g, yield 82%). ^{1}H NMR δ_H (ppm) (400 MHz, $CDCl_3$): 9.03-9.02 (d, 1H), 8.83-8.82 (d, 1H), 8.68-8.67(d, 1H), 8.59-8.56 (m, 1H), 8.39-8.36 (d, 1H).

Polymerization of PHATMA

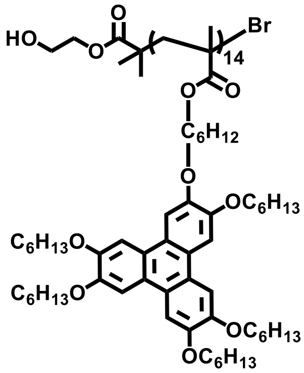

CuBr (5.8 mg, 0.04 mmol), PMDETA (8.4 μL, 0.04 mmol), 6-[3,6,7,10,11-pentakis(hexyloxy)-2-oxytriphenylene]hexyl methacrylate (548 mg, 0.6 mmol), HEBiB (3.0 μL, 0.02 mmol) and 2 mL 1,4-dioxane were introduced into a Schlenk tube and degassed with three freeze-pump-thaw cycles. Subsequently, the polymerization solution was heated at 80 °C for 6 h under a nitrogen atmosphere with vigorous stirring. The reaction mixture was purified *via* column chromatography with PE/ethyl acetate (silica gel, 10/1, volume ratio) as the eluent first to remove the residual trace monomer. Then the polymer was eluted with ethyl acetate. The concentrated crude product was further purified *via* repeated precipitations from THF solution into MeOH, and dried under reduced pressure. A white solid was obtained as the final product (350 mg, yield 57.6%).

Supplementary Note 1. AFM-IR.

The IR laser energy was measured before the light entered the polarizer, since turning the polarizer changes not only the polarization but also the actual energy reaching the sample.[3, 4] A correction factor must be introduced to account for the energy difference between the two polarization directions so that the difference in the two corrected polarized spectra can be solely attributed to molecular orientation. Amorphous polymethyl methacrylate (PMMA) was chosen as the correction sample. The correction factor was defined as the area ratio of the perpendicular (90°) to the parallel (0°) for 1730 cm^{-1}, corresponding to the absorbance of carbonyl groups. The IR laser power level for this particular experiment was 1.78%, and the average correction factor determined was 0.77.

Supplementary Note 2. Experimental procedure to determine the doping ratio

To check if the dopant molecules all formed EDA complexes with HAT, the doped micelles were first dialyzed against 2-PrOH for 7 days (solvent was changed 14 times) to remove any possible free TNF molecules. Subsequently, the micellar solution was dried under nitrogen flow and then redispersed in THF to dissolve both diblock copolymer and TNF at the molecular level. The THF solution was then dialyzed against THF again and the solution outside the dialysis tube was collected and concentrated. The TNF content was determined with FL spectrometry *via* comparison with the standard curve (Supplementary Figure 38).

Supplementary Note 3. Persistence length calculation.[5]

To analyze the persistence lengths of the doped and undoped fibrils, FibreApp and analysis methods from the literature were adopted.[5] For the persistence length analysis, the black and red traces were used, which were the fibrils tracked with the software in the image, as shown in Supplementary Figure 57. Mean-squared end-to-end distance (MSED) for a worm-like chain (WLC) model was dependent on the internal contour length in two dimensions, which was calculated using eq. S1,

$$\langle R^2 \rangle = 4\lambda[l - 2\lambda(1-e^{-l/2\lambda})] \quad \text{(S1)}$$

where λ is the persistence length, and R is the direct distance between any pair of segments along a contour separated by arc length l. In Supplementary Figure 57, the end-to-end distance method showed good fitting results for the doped fibrils (black) and the undoped fibrils (red).

Supplementary Note 4. The calculation of the fraction of surviving seeds

Since the mass of polymer *m* in solution was kept unchanged before and after the self-seeding experiment, the length of micelles L_n can be related to the total number of micelles *N* by equation (S2), if we assume i) the concentration of free polymer chains at r.t. is negligible, ii) the mass of polymer per unit length *M* remains constant.

$$L_n = \frac{m}{M_L N} \qquad \text{(S2)}$$

$$\text{Percentage of surviving seeds} = \frac{L_{\text{seed}}}{L_n} \times 100\% \qquad \text{(S3)}$$

Based on equations S2 and S3, we could calculate the fraction of seeds that survived at each annealing temperature by taking into account the initial length of the fragments and the final length after annealing. The results are summarized in Figure 5c. In the range of 60-72 ^{o}C, the fraction of surviving seeds decreased exponentially with increasing temperature, a key characteristic of a self-seeding process.[6, 7]

Supplementary Note 5. “Self-seeding” process.

As shown in Figure 5(c), the length of the micelles increased exponentially with the annealing temperature. Subsequently, the data in Figure 5(c) was replotted in Supplementary Figure 62 as $ln(L_n)$ *versus* 1000/T. In the range of 60-72 ºC, the data fitted well to the Arrhenius-type plot. Setting the slope of the plot equal to -E/R, where R is the gas constant, we calculate an activation energy (E) value of 35.8 kJ/mol for the self-seeding process (Supplementary Figure 62), which was significantly lower than those from similar systems with much stronger driving forces.[8, 9] Since micelle elongation operates under thermodynamic rather than kinetic control, this E value should be related to thermodynamic factors, for example, the enthalpy of dissolution of the micelles in 2-PrOH. It is noteworthy that this value is much smaller than that reported for the LC poly(2-(perfluorooctyl)ethyl methacrylate (PFMA) system (69 kJ/mol) in our previous study.[9]

From DSC traces (Supplementary Figure 21), the LC phase transition enthalpy is 2.57 J/g for PHATMA, smaller than those from other liquid crystalline block copolymers (such as PFMA, 2.8 J/g). It is also much smaller than the melting enthalpy values of crystalline polymers, such as polyethylene (30-69 J/g),[10] poly(ferrocenyldimethylsilane) (PFS, 7.7 and 14.7 J/g).[11] This lower phase-transition energy is consistent with our finding mentioned above that the enthalpy of dissolution of these micelles (35.8 kJ/mol) in our study is smaller than that in PFMA systems (69 kJ/mol)[9] and PFS systems (149 kJ/mol)[8].

Supplementary Figures

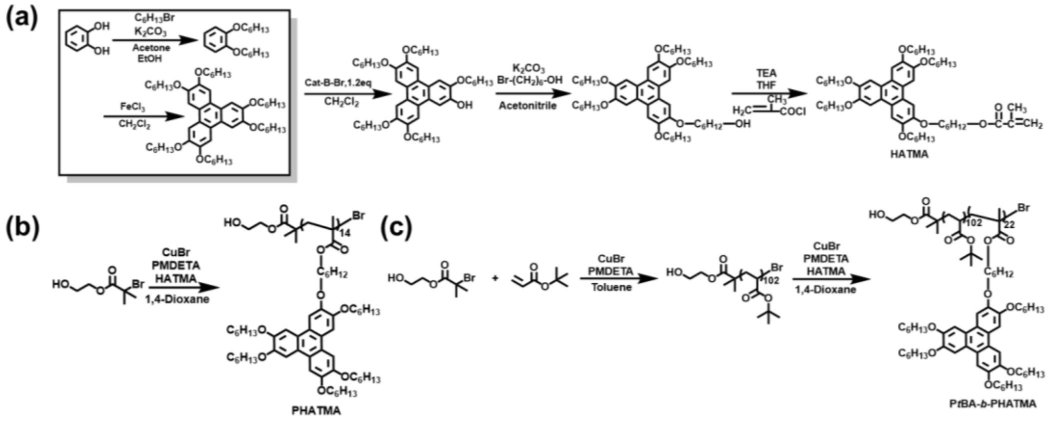

Supplementary Figure 1. Synthetic routes of the monomers and polymers. Synthetic routes of the (a) HATMA monomer, (b) PHATMA homopolymer, and (c) P*t*BA-*b*-PHATMA diblock copolymer.

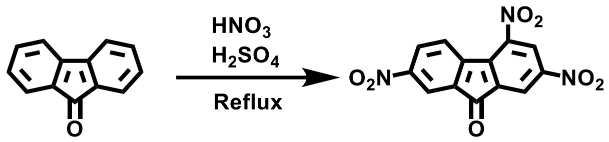

Supplementary Figure 2. Synthetic route of TNF.

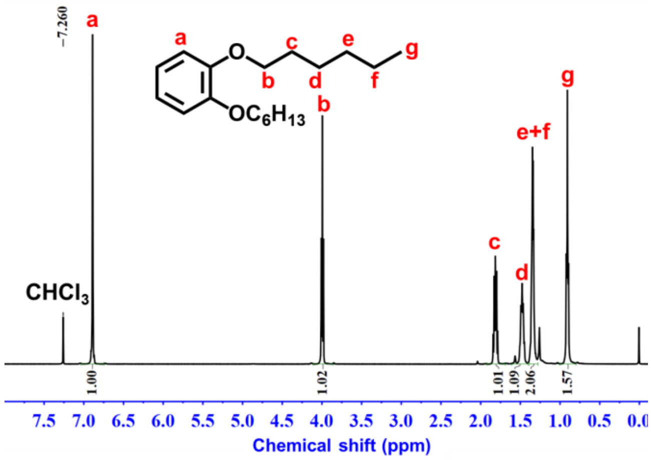

Supplementary Figure 3. ^{1}H NMR spectrum of precursor. ^{1}H NMR spectrum of the 1,2-di(hexyloxy)benzene. $CDCl_3$ was used as the solvent.

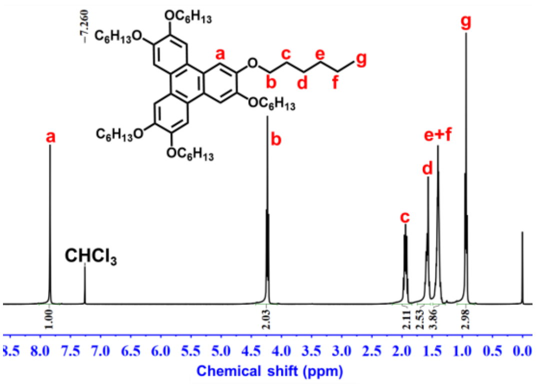

Supplementary Figure 4. ^{1}H NMR spectrum of the precursor of monomer. ^{1}H NMR spectrum of the 2,3,6,7,10,11-hexakis(hexyloxy)triphenylene. $CDCl_3$ was used as the solvent.

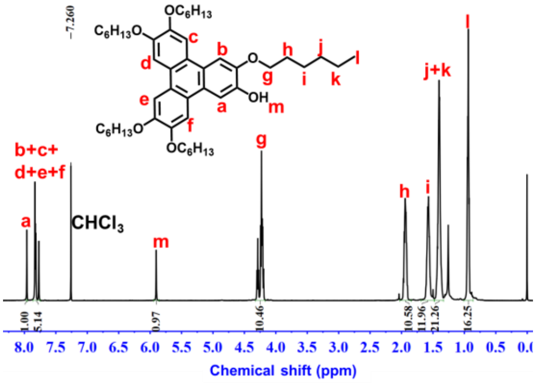

Supplementary Figure 5. ^{1}H NMR spectrum of the precursor of monomer. ^{1}H NMR spectrum of the 2-hydroxy-3,6,7,10,11-penta(hexyloxy)triphenylene. $CDCl_3$ was used as the solvent.

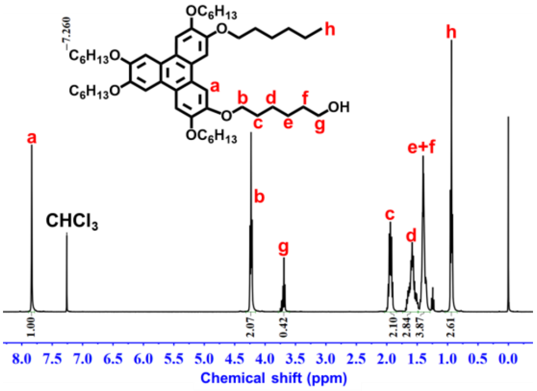

Supplementary Figure 6. ^{1}H NMR spectrum of the precursor of monomer. ^{1}H NMR spectrum of the 2-(6-hydroxylhexyloxy)-3,6,7,10,11-pentakis(hexyloxy)triphenylene. $CDCl_3$ was used as the solvent.

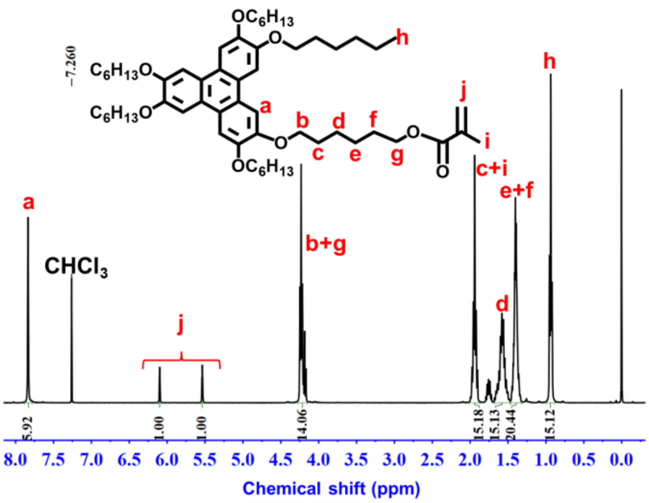

Supplementary Figure 7. ^{1}H NMR spectrum of monomer. ^{1}H NMR spectrum of the 6-[3,6,7,10,11-pentakis(hexyloxy)-2-oxytriphenylene]hexyl methacrylate. $CDCl_3$ was used as the solvent.

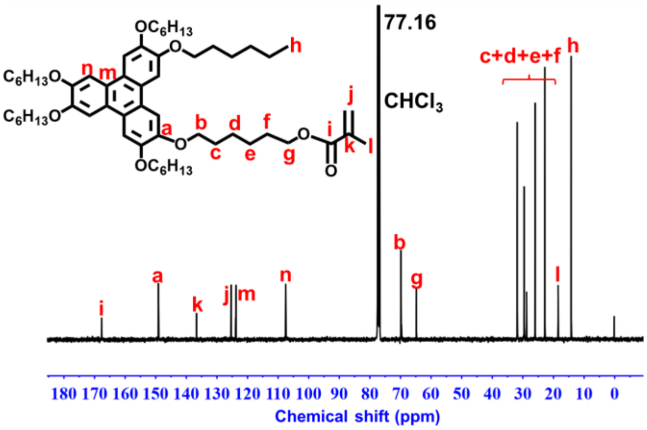

Supplementary Figure 8. ^{13}C NMR spectrum of monomer. ^{13}C NMR spectrum of the 6-[3,6,7,10,11-pentakis(hexyloxy)-2-oxytriphenylene]hexyl methacrylate. $CDCl_3$ was used as the solvent.

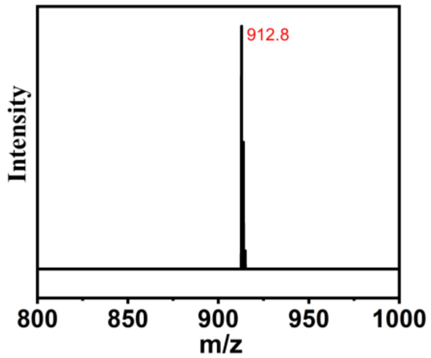

Supplementary Figure 9. MALDI-TOF-MS spectrum of the 6-[3,6,7,10,11-pentakis(hexyloxy)-2-oxytriphenylene]hexyl methacrylate.

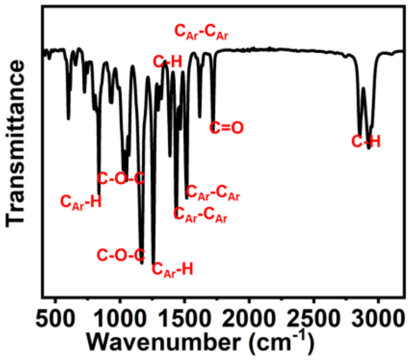

Supplementary Figure 10. FT-IR spectrum of the 6-[3,6,7,10,11-pentakis(hexyloxy)-2-oxytriphenylene]hexyl methacrylate.

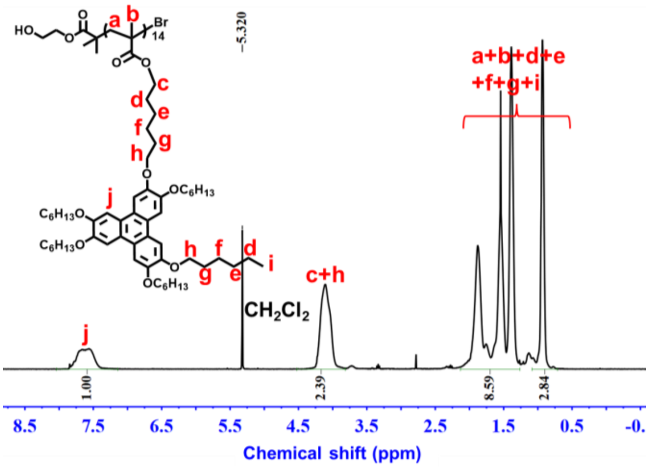

Supplementary Figure 11. ^{1}H NMR spectrum of the polymer. ^{1}H NMR spectrum of the PHATMA homopolymer. CD_2Cl_2 was used as the solvent.

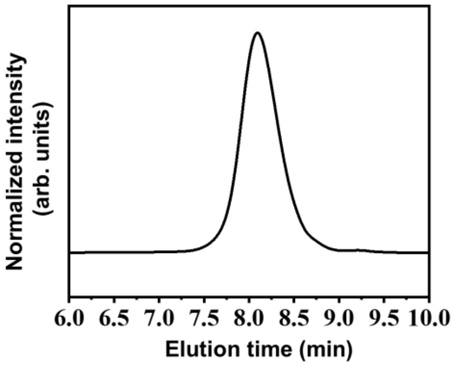

Supplementary Figure 12. GPC trace of the PHATMA homopolymer.

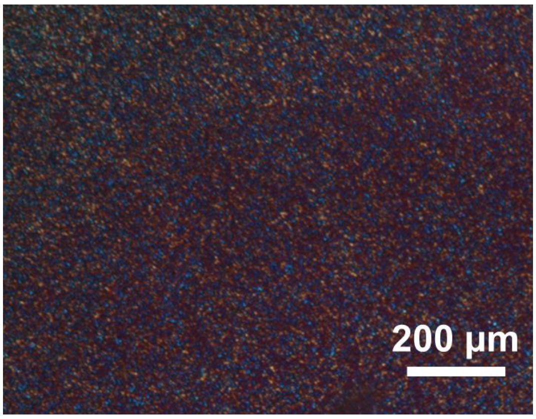

Supplementary Figure 13. POM image of the textures of the PHATMA homopolymer.

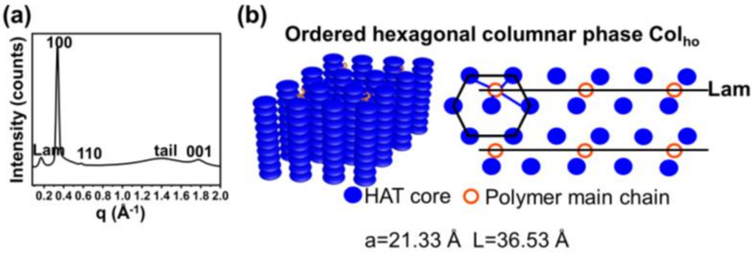

Supplementary Figure 14. Molecular packing of the HAT disks. (a) WAXS spectrum and (b) schematic illustrations of packings of HAT disks inside PHATMA homopolymer bulk.

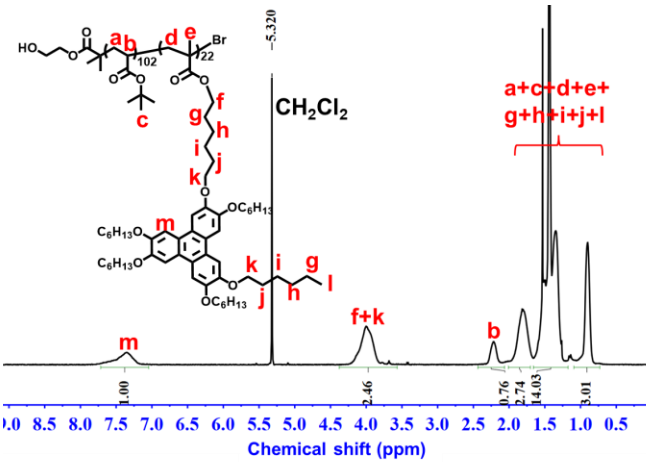

Supplementary Figure 15. ^{1}H NMR spectrum of the diblock copolymer. ^{1}H NMR spectrum of the P*t*BA-b-PHATMA. CD_2Cl_2 was used as the solvent.

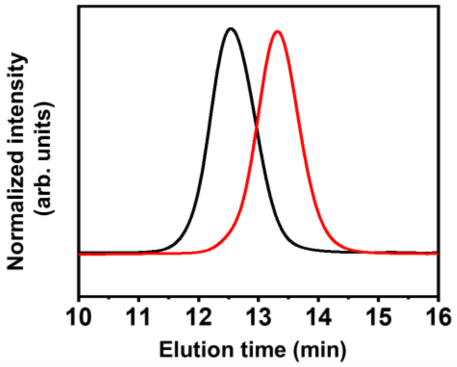

Supplementary Figure 16. GPC analysis of the polymers. GPC traces of the P*t*BA homopolymer (red) and P*t*BA-*b*-PHATMA diblock copolymer (black).

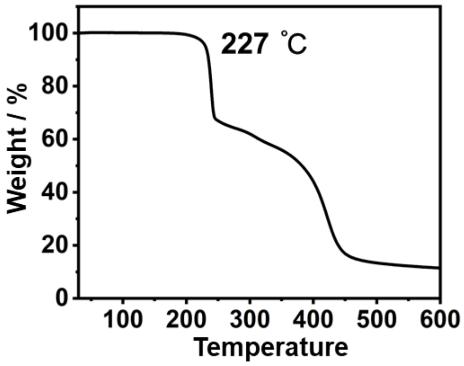

Supplementary Figure 17. TG trace of the P*t*BA-*b*-PHATMA diblock copolymer.

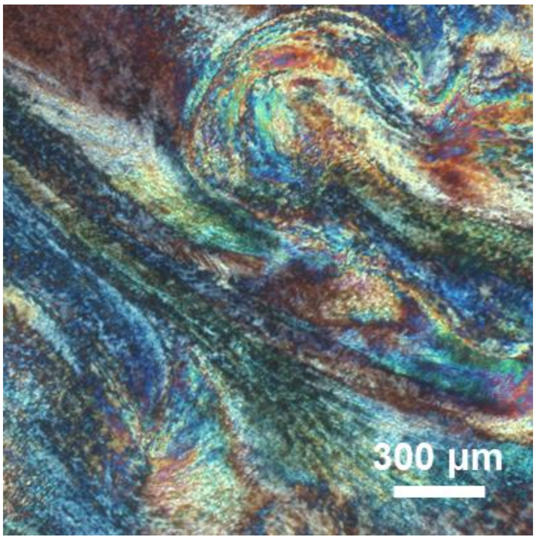

Supplementary Figure 18. POM image of the textures of the P*t*BA-*b*-PHATMA diblock copolymer.

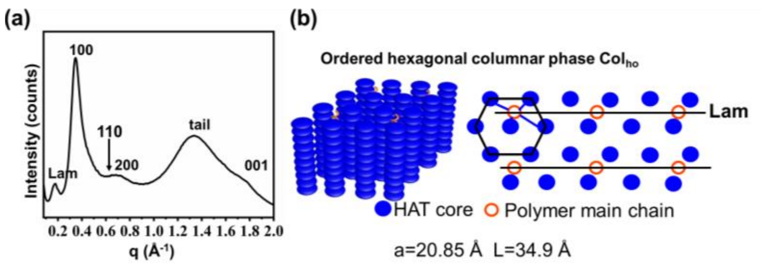

Supplementary Figure 19. Molecular packing of the HAT disks. (a) WAXS spectrum and (b) schematic illustrations of the structure of the bulk sample of the diblock copolymer.

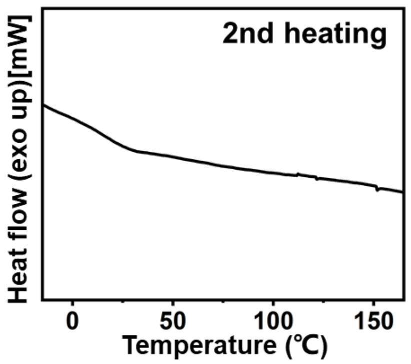

Supplementary Figure 20. DSC trace of the bulk P*t*BA-*b*-PHATMA diblock copolymer.

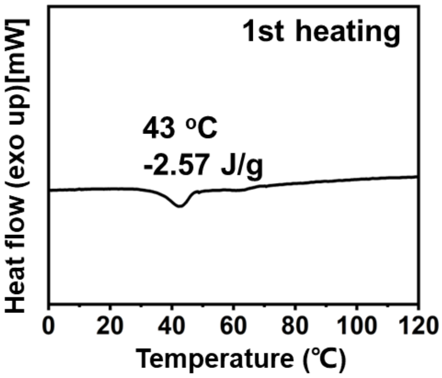

Supplementary Figure 21. DSC trace of the bulk P*t*BA-*b*-PHATMA diblock copolymer after annealing.

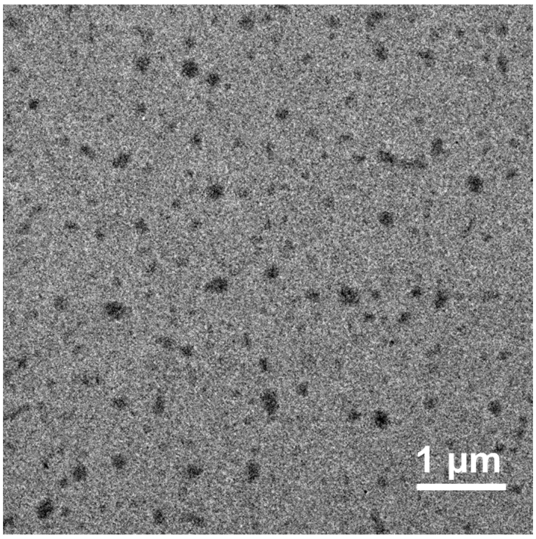

Supplementary Figure 22. TEM image of the assemblies from P*t*BA-*b*-PHATMA diblock copolymer at 80 °C in 2-PrOH.

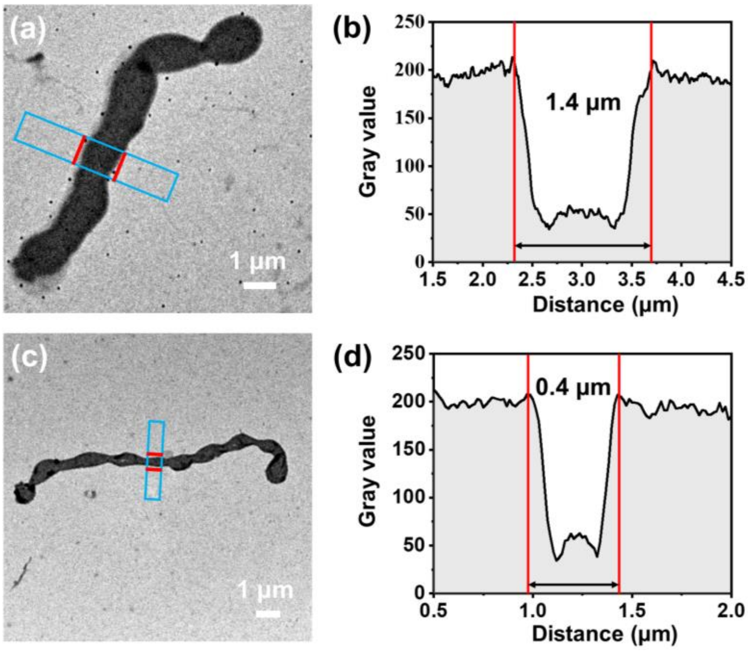

Supplementary Figure 23. Analysis of the hollow tubular nanostructures. (a, c) TEM images and (b, d) the corresponding grayscale analyses of the tubular structures by dispersing the P*t*BA-*b*-PHATMA in 2-PrOH at 80 °C for 1 h and after the solution reached r.t.

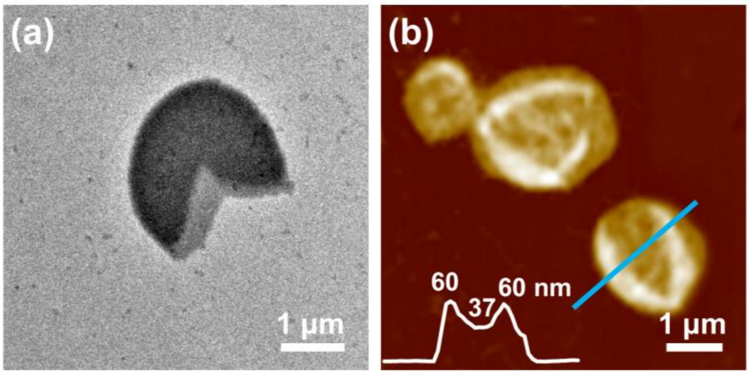

Supplementary Figure 24. Analysis of the hollow vesicular nanostructures. (a) TEM and (b) AFM topographic images of the vesicular structures by dispersing the P*t*BA-*b*-PHATMA in 2-PrOH at 80 ℃ for 1 h and cooling down to r.t. within 10 h storage.

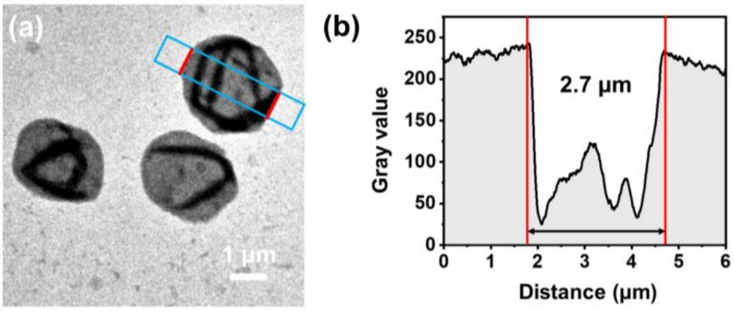

Supplementary Figure 25. Analysis of the hollow vesicular nanostructures. (a) TEM images and (b) the corresponding grayscale analyses of the vesicular structures by dispersing the P*t*BA-*b*-PHATMA in 2-PrOH at 80 ℃ for 1 h and cooling down to r.t. within 10 h storage.

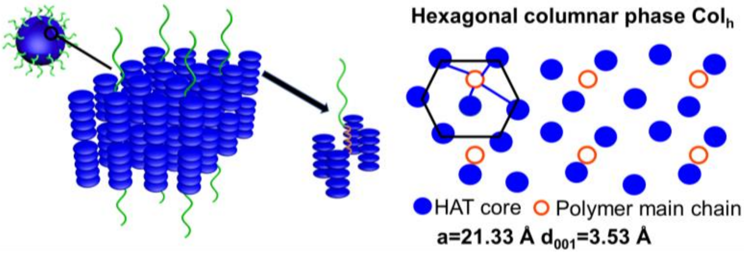

Supplementary Figure 26. Molecular packing of the HAT disks. Schematic illustrations of the molecular packing of mesogens in the membrane of the vesicles from P*t*BA-*b*-PHATMA.

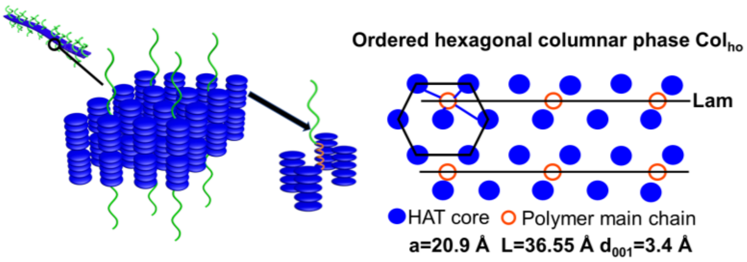

Supplementary Figure 27. Molecular packing of the HAT disks. Schematic illustration of the molecular packing of mesogens in the core of the fibrils from P*t*BA-*b*-PHATMA.

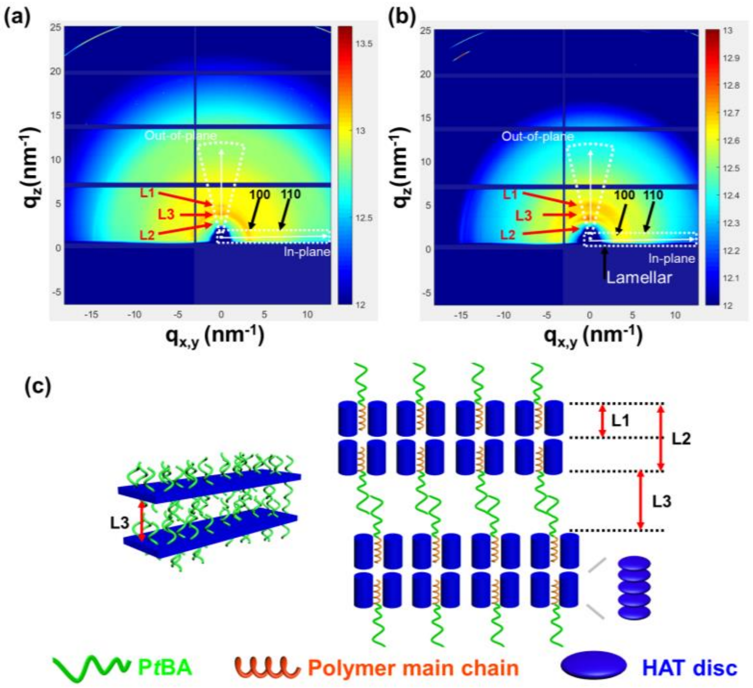

Supplementary Figure 28. GI-SAXS characterizations. GI-WAXS spectra of the (a) vesicle and (b) fibril structures. (c) A schematic cartoon of the packing geometry.

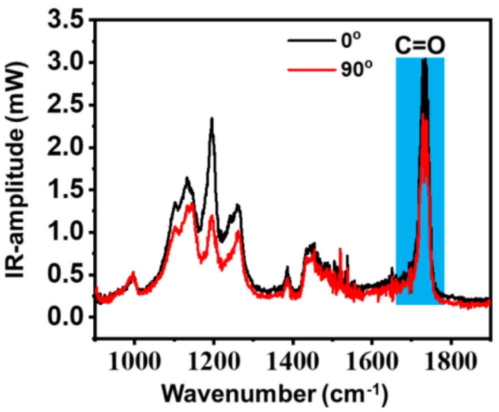

Supplementary Figure 29. Polarized AFM-IR spectra of PMMA.

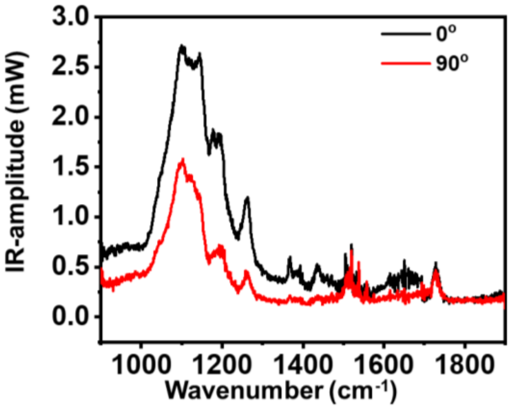

Supplementary Figure 30. Polarized AFM-IR spectra of the thin fibrils without correction.

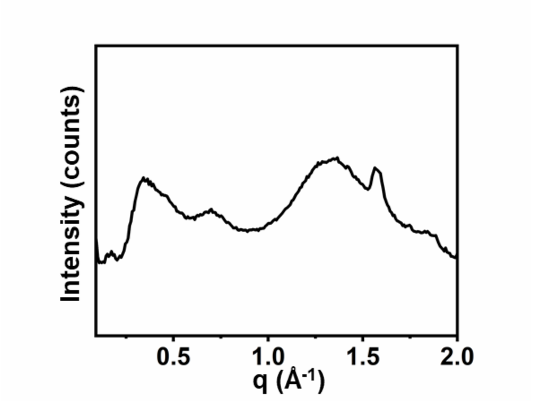

Supplementary Figure 31. WAXS analysis. WAXS spectrum of the aggregates produced in the 2-PrOH solution of P*t*BA-*b*-PHATMA by directly taking the hot solution out of oil bath and cooling it in air.

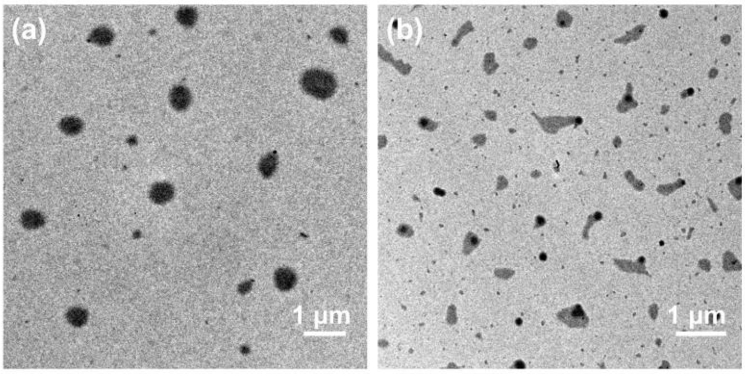

Supplementary Figure 32. Aggregates from different protocols. TEM images of the aggregates from the P*t*BA-*b*-PHATMA in 2-PrOH solution obtained by (a) quenching the solution from 80 °C to r.t. within 1 minute, and (b) then storing for 6 months at r.t.

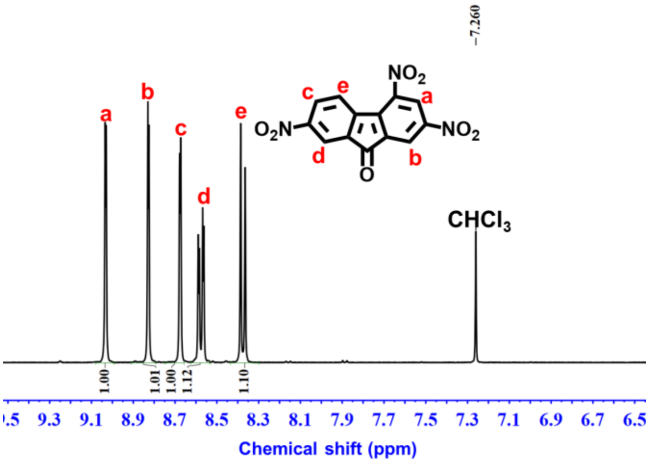

Supplementary Figure 33. ^{1}H NMR spectrum. ^{1}H NMR spectrum of TNF. $CDCl_3$ was used as the solvent.

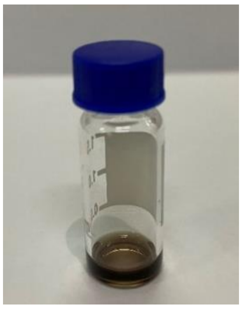

Supplementary Figure 34. Photographic image of the EDA THF solution. The picture was taken immediately after adding TNF into the THF solution of PHATMA.

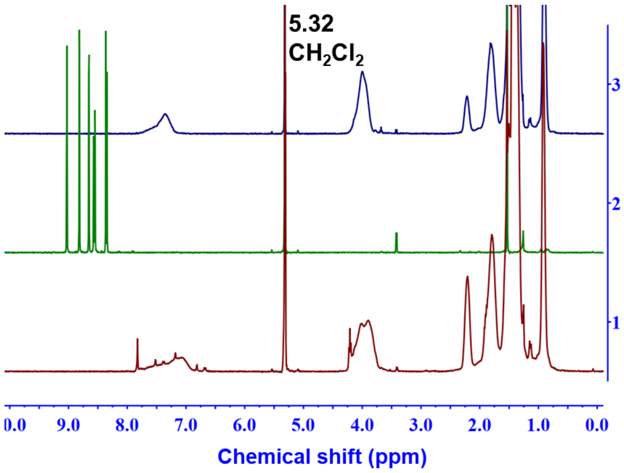

Supplementary Figure 35. The formation of EDA. ^{1}H NMR spectra of (1) EDA complexes, (2) TNF, and (3) the P*t*BA-*b*-PHATMA diblock copolymer. CD_2Cl_2 was used as the solvent. New peaks emerged at 7.83, 7.52, 7.39-7.37, 7.18, 6.81, 6.69-6.67, and 4.23-4.19 ppm in the ^{1}H NMR spectra after the mixing, suggesting the formation of EDA complex between the HAT aromatic core and the TNF.

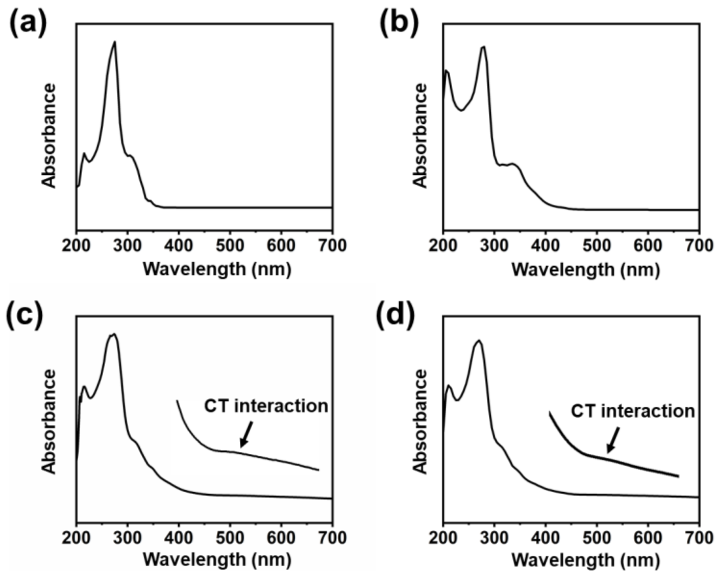

Supplementary Figure 36. The demonstration of EDA formation. UV-vis absorbance spectra of (a) the P*t*BA-*b*-PHATMA diblock copolymer, (b) TNF, (c) EDA complexes ($r = 0.5$), and (d) EDA complexes ($r = 1$) in the 2-PrOH solution. New peaks emerged at 525 nm in the UV-vis spectra confirming the formation of EDA.

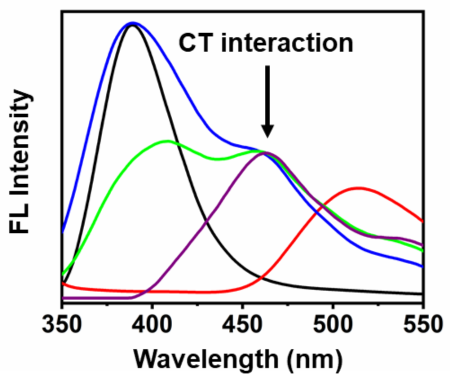

Supplementary Figure 37. The demonstration of EDA formation by FL spectra. FL spectra of the 2-PrOH solutions of P*t*BA-*b*-PHATMA (black), TNF (red), doped P*t*BA-b-PHATMA with $r = 0.2$ (blue), $r = 0.5$ (green), and $r = 1$ (purple). New peaks emerged at 462 nm in the FL spectra confirming the formation of EDA.

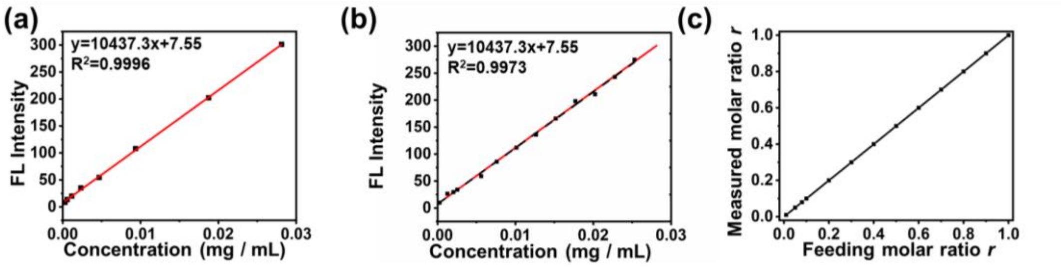

Supplementary Figure 38. Content of TNF. The plot of (a) the standard curve of the FL intensity versus the concentration of TNF in THF, (b) the experimental data points (black points) fitted (dashed black line) in the standard curve (solid red line), and (c) comparison between doping molar ratio and feeding molar ratio.

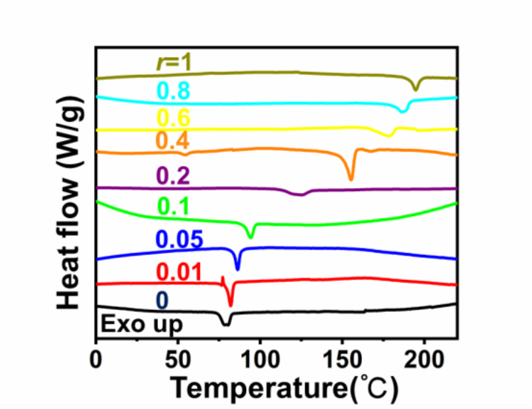

Supplementary Figure 39. DSC traces of the bulk PHATMA homopolymer with different doping ratios.

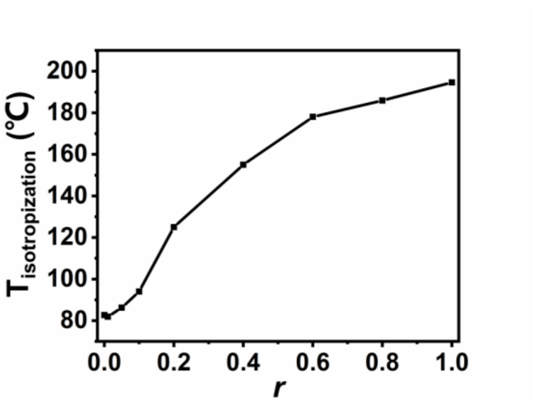

Supplementary Figure 40. Variations of the phase-transition temperatures of PHATMA *versus* doping ratio.

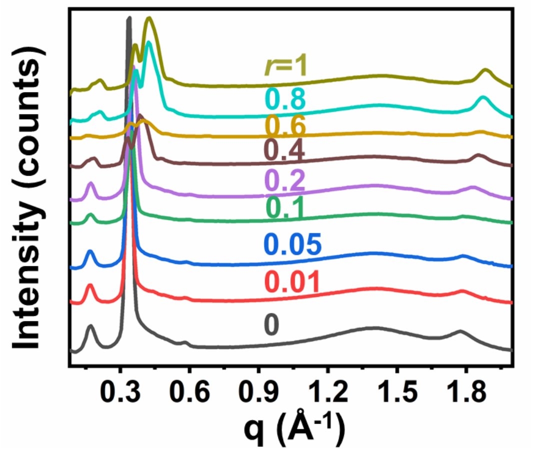

Supplementary Figure 41. WAXS spectra of the bulk sample of PHATMA homopolymer with varying doping ratio.

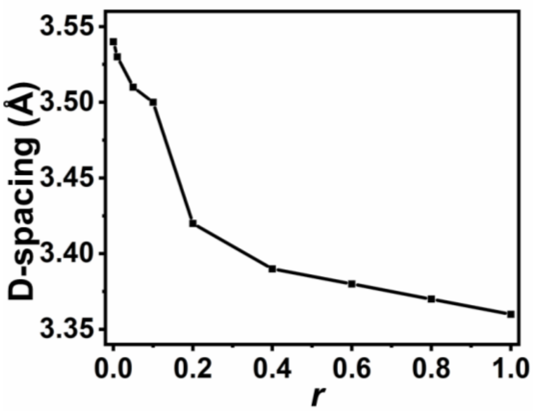

Supplementary Figure 42. Variations of the *d*-spacing distances between discotic mesogens from PHATMA versus doping ratio.

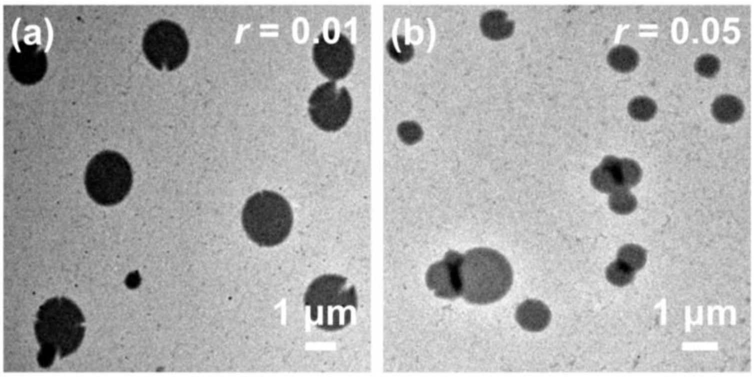

Supplementary Figure 43. Morphologies of self-assemblies with different doping ratios. TEM images of the self-assembled structures by dispersing the doped P*t*BA-*b*-PHATMA with various *r*-values in 2-PrOH at 80 °C for 1 h and cooling down to r.t. naturally: (a) $r = 0.01$ and (b) $r = 0.05$.

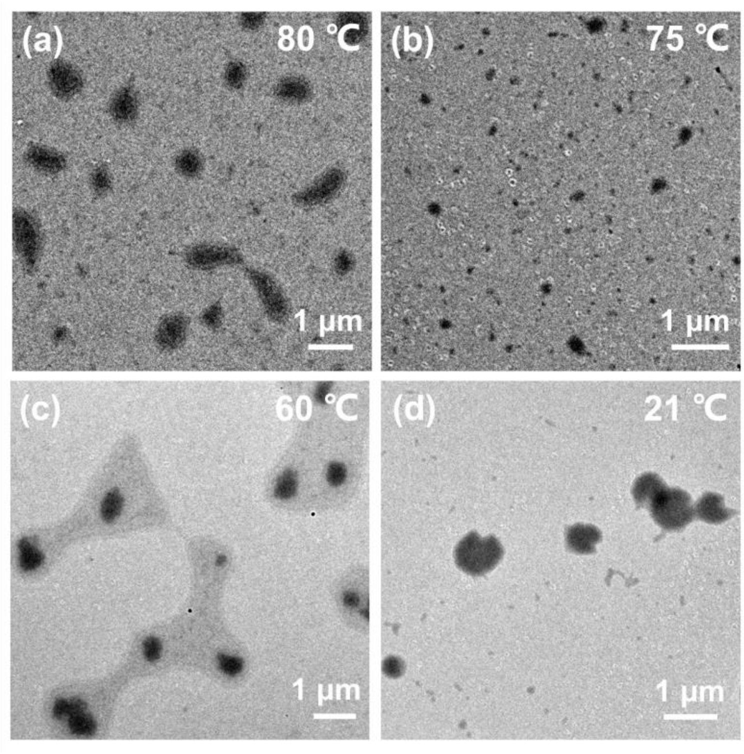

Supplementary Figure 44. Self-assembled structures during cooling process. TEM images of the self-assembled structures by dispersing the doped P*t*BA-*b*-PHATMA (*r* = 0.01) in 2-PrOH at 80 °C for 1 h and cooling down to r.t. naturally: (a) dispersed individual chains, (b) small spheres, (c) big spheres, (d) vesicular structures.

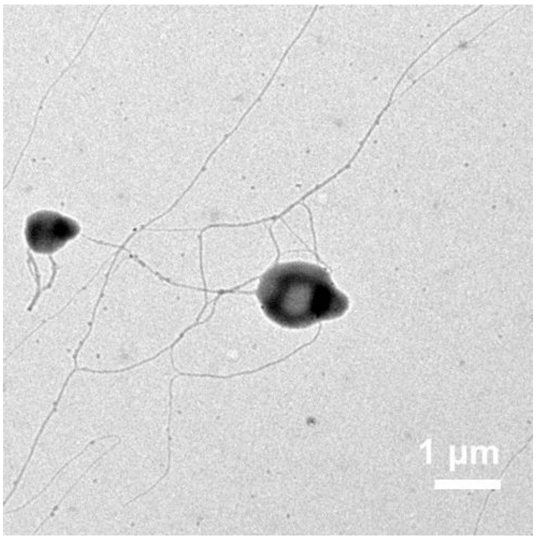

Supplementary Figure 45. TEM image of the assemblies from the doped P*t*BA-*b*-PHATMA (*r* = 0.01) in 2-PrOH after one-year storage at r.t.

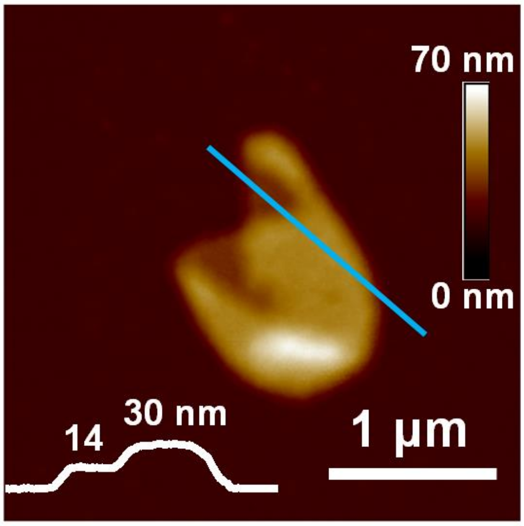

Supplementary Figure 46. Doped vesicle. AFM topographic image of the self-assembled structures by dispersing the doped P*t*BA-*b*-PHATMA ($r = 0.1$) in 2-PrOH at 80 °C for 1 h and cooling down to r.t. naturally.

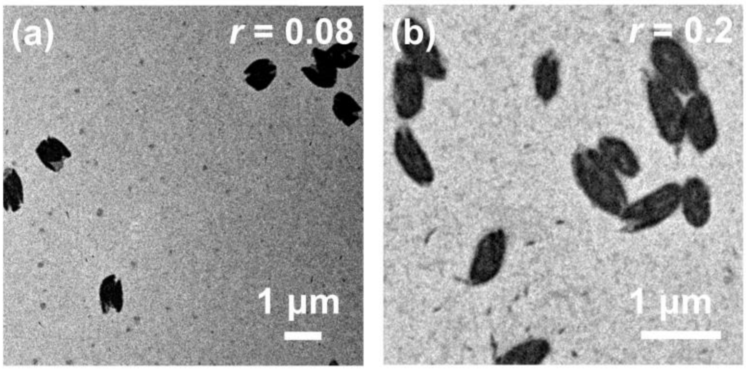

Supplementary Figure 47. Morphologies of self-assemblies with different doping ratios. TEM images of the self-assembled structures by dispersing the doped P*t*BA-*b*-PHATMA with various *r*-values in 2-PrOH at 80 °C for 1 h and cooling down to r.t. naturally: (a) $r = 0.08$ and (b) $r = 0.2$.

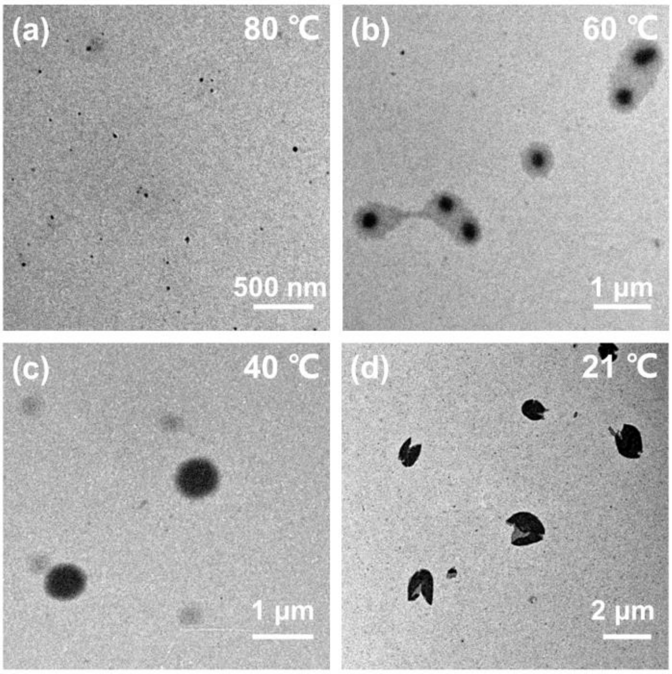

Supplementary Figure 48. Self-assembled structures during cooling process. TEM images of the self-assembled structures by dispersing the doped P*t*BA-*b*-PHATMA (r = 0.1) in 2-PrOH at 80 °C for 1 h and cooling down to r.t. naturally: (a) small spheres, (b) big spheres, (c) spherical vesicles, (d) ellipsoidal vesicles.

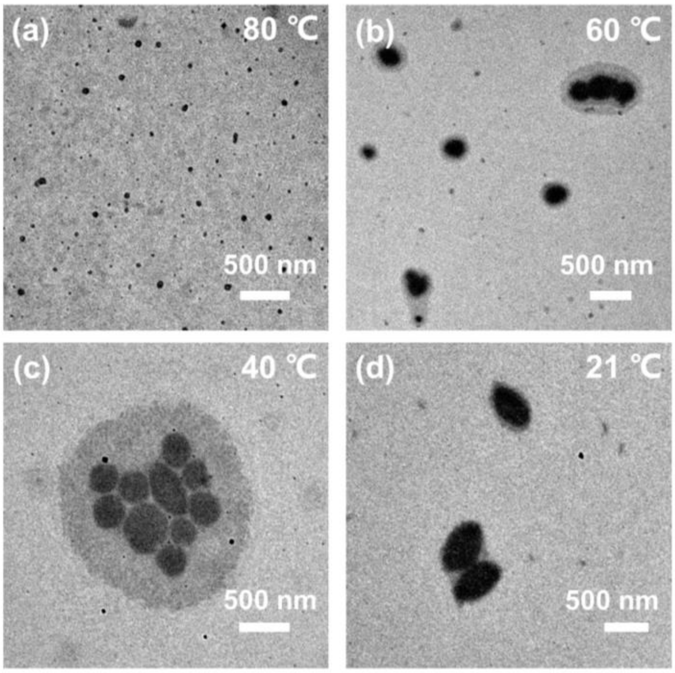

Supplementary Figure 49. Self-assembled structures during cooling process. TEM images of the self-assembled structures by dispersing the doped P*t*BA-*b*-PHATMA (r = 0.2) in 2-PrOH at 80 °C for 1 h and cooling down to r.t. naturally: (a) small spheres, (b) big spheres, (c) spherical vesicles, (d) ellipsoidal vesicles.

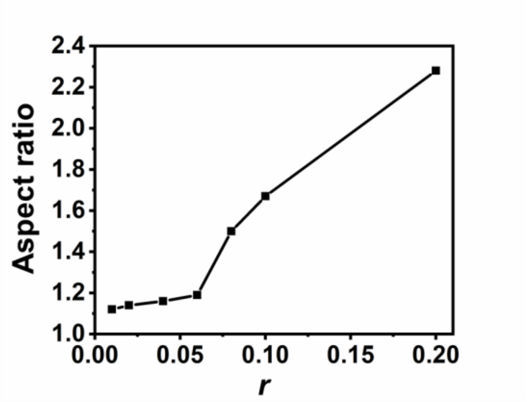

Supplementary Figure 50. Variations of the aspect ratio of the vesicular assemblies versus doping ratios.

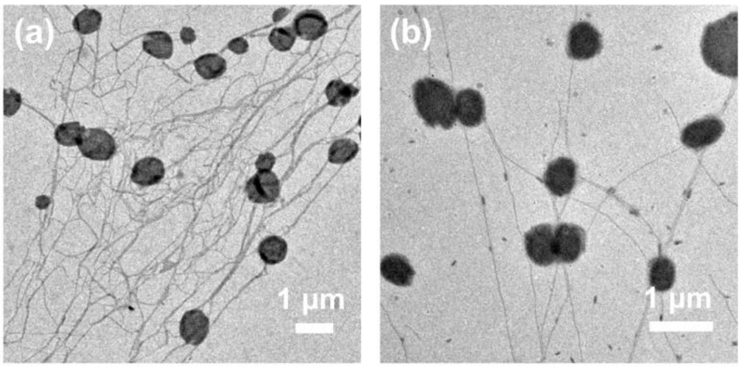

Supplementary Figure 51. Morphologies of self-assemblies with different doping ratios. TEM image of the assemblies from the doped P*t*BA-*b*-PHATMA in 2-PrOH after one-year storage at r.t.: (a) $r = 0.1$ and (b) $r = 0.2$.

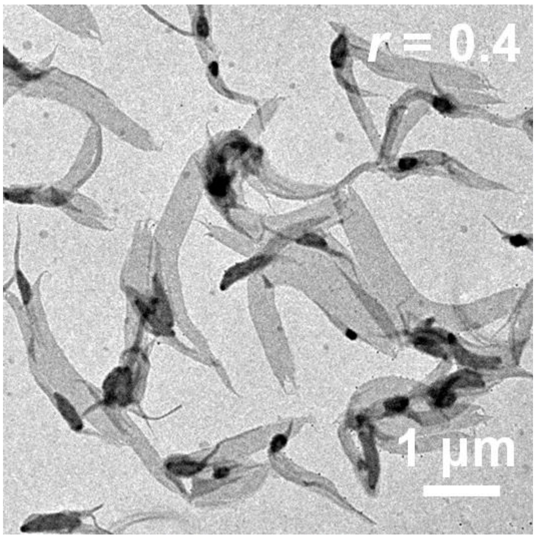

Supplementary Figure 52. Self-assemblies from doped diblock copolymer. TEM image of the self-assembled structures by dispersing the doped P*t*BA-*b*-PHATMA (r = 0.4) in 2-PrOH at 80 °C for 1 h and cooling down to r.t. naturally.

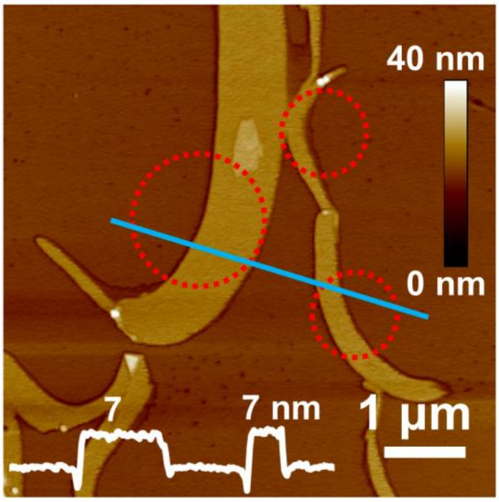

Supplementary Figure 53. Belt-like structures. AFM topographic images of the self-assembled structures by dispersing the doped P*t*BA-*b*-PHATMA (r = 0.5) in 2-PrOH at 80 °C for 1 h and cooling down to r.t. naturally.

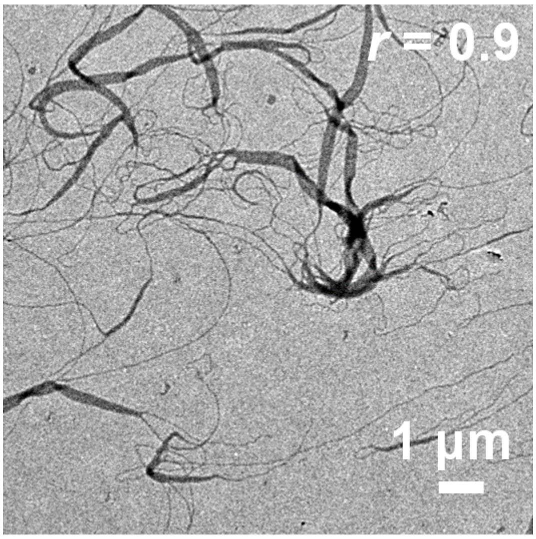

Supplementary Figure 54. Self-assemblies from doped diblock copolymer. TEM images of the self-assembled structures by dispersing the doped P*t*BA-*b*-PHATMA (r = 0.9) in 2-PrOH at 80 °C for 1 h and cooling down to r.t. naturally.

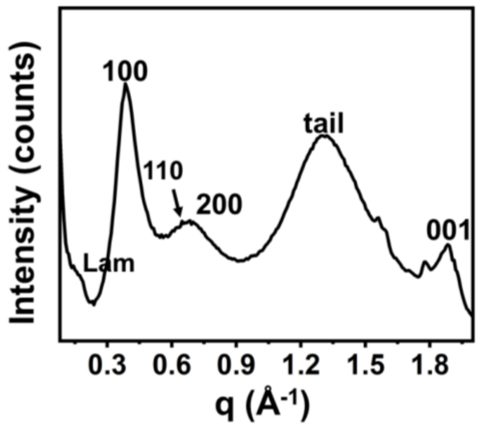

Supplementary Figure 55. WAXS spectrum of the dried assemblies with *r* = 1.

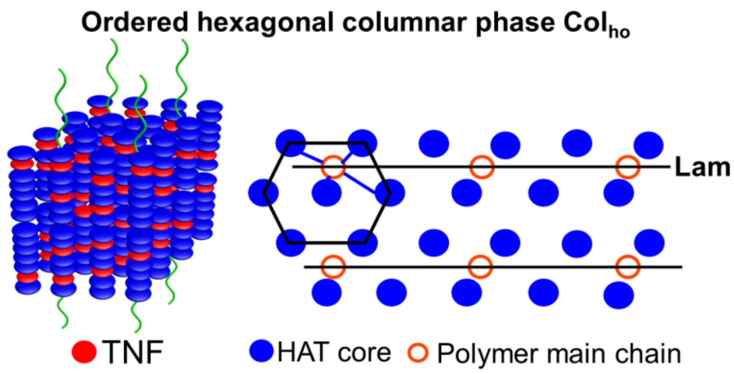

Supplementary Figure 56. Molecular packing of the HAT disks. Schematic illustrations of the molecular packings of the mesogens by dispersing the P*t*BA-*b*-PHATMA and TNF in 2-PrOH at 80 °C for 1 h and cooling down to r.t.

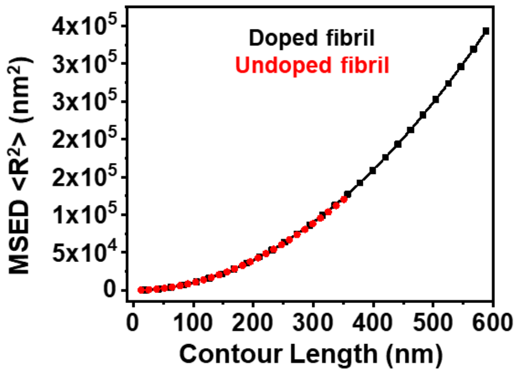

Supplementary Figure 57. Plots of MSED versus internal contour length of the doped fibrils and the undoped fibrils.

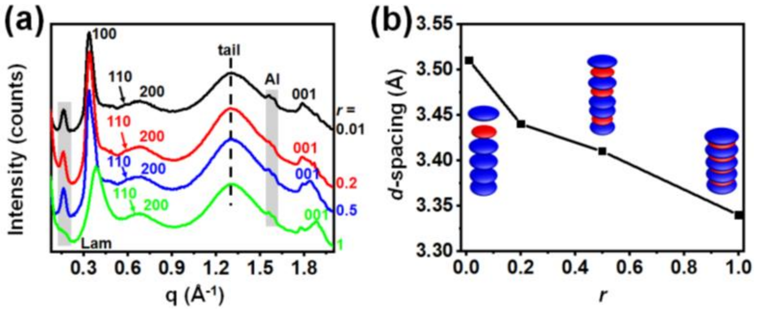

Supplementary Figure 58. Analysis of the HAT disk packings after doping with TNF. Comparison of the (a) WAXS spectra, and (b) *d*-spacing of the assemblies with various *r*.

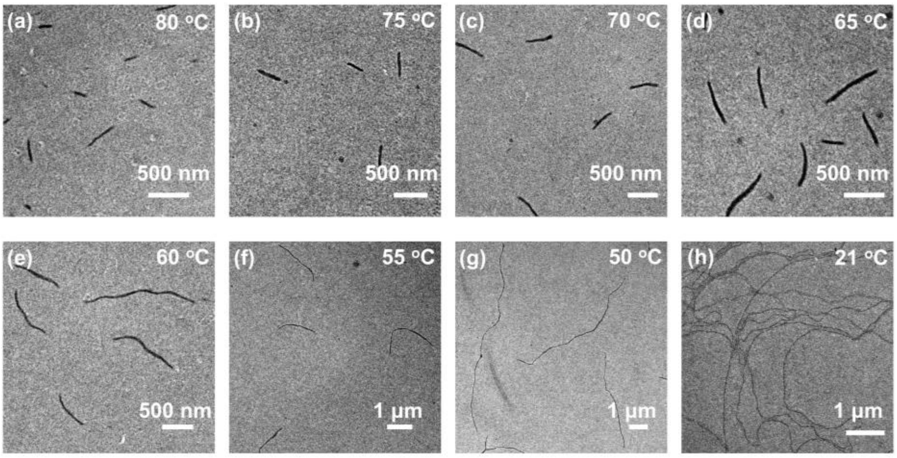

Supplementary Figure 59. Self-assembled structures during cooling process. TEM images of the doped P*t*BA$_{102}$-*b*-PHATMA$_{22}$ micelles (0.1 mg/mL) with r = 1 formed during the cooling process at (a) 80 ºC, (b) 75 ºC, (c) 70 ºC, (d) 65 ºC, (e) 60 ºC, (f) 55 ºC, (g) 50 ºC, and (h) 21 ºC. The length information of all the samples is summarized in Supplementary Table 3.

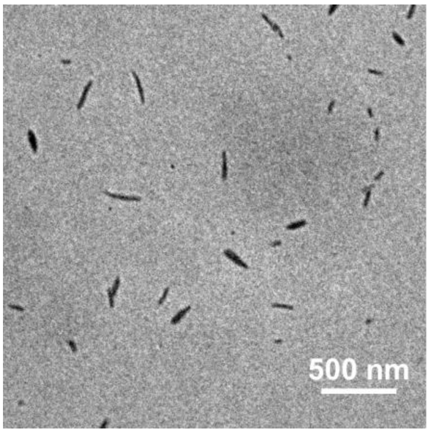

Supplementary Figure 60. The seeds obtained by ultrasonication of the doped fibrils solution (*r*=1) in 2-PrOH at 0 °C for 4 h.

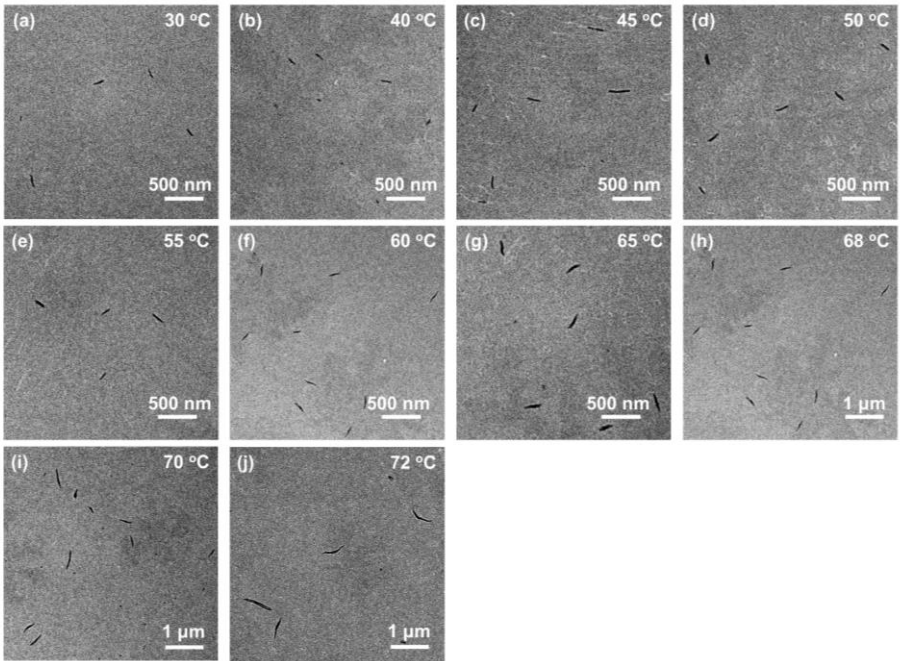

Supplementary Figure 61. Self-assembled structures during cooling process. TEM images of the fibrillar micelles obtained by annealing the 2-PrOH solution of seeds (0.005 mg/mL) at (a) 30 ºC, (b) 40 ºC, (c) 45 ºC, (d) 50 ºC, (e) 55 ºC, (f) 60 ºC, (g) 65 ºC, (h) 68 ºC, (i) 70 ºC, and (j) 72 ºC. The length information of all the samples is summarized in Supplementary Table 4.

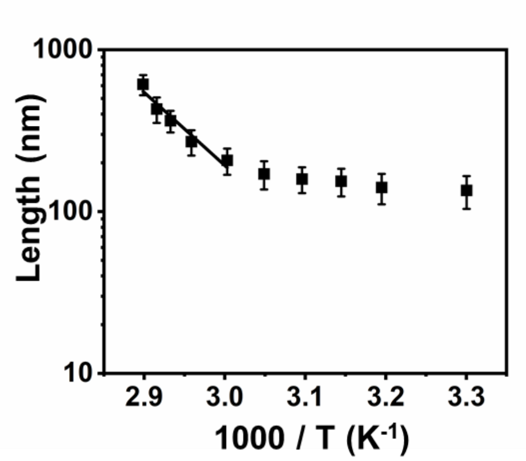

Supplementary Figure 62. Analysis of the micelle length. Semilogarithmic plots of micelle length L_n versus 1000/T [K^{-1}], where T is the dissolution temperature (from 30 ºC to 75 ºC). The length information of all the samples is summarized in Supplementary Table 4. Error bars represent mean ± standard deviation, n ≥ 200. The straight line represents the best fit for the points of T to $ln(L_n)$ = A-E/RT (E = 35.8 kJ/mol), where A is a constant and R is the gas constant. Errors bars are standard deviations of the lengths.

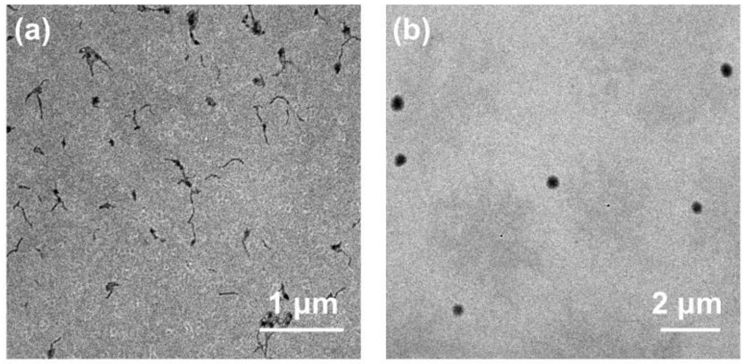

Supplementary Figure 63. Self-assembled structures from different protocols. TEM images of (a) the seeds obtained by ultrasonication of the undoped fibrils 2-PrOH solution at 0 °C for 4 h, and (b) the aggregates obtained by annealing the 2-PrOH solution of these seeds (0.005 mg/mL) at 65 °C.

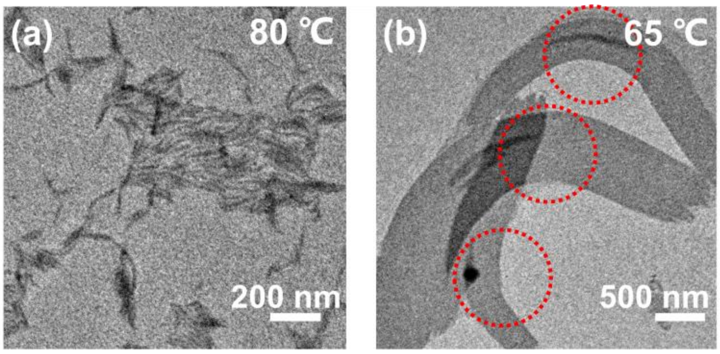

Supplementary Figure 64. Self-assembled structures during cooling process. TEM images of the assemblies from the doped P*t*BA-*b*-PHATMA (*r* = 0.5) in 2-PrOH at (a) 80 °C and (b) 65 °C.

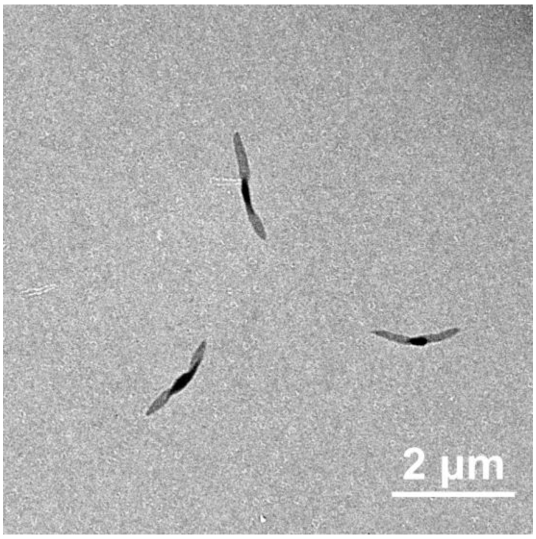

Supplementary Figure 65. Seed-grown belt-like structures. TEM images of the uniform belt-like structures formed by thermally annealing the fully-doped fibrillar seeds and belt fragments together with M_{belt}/M_{seed} = 10 in 2-PrOH at 70 °C for 1 h and cooling down to r.t. naturally (seed concentration = 0.002 mg/mL).

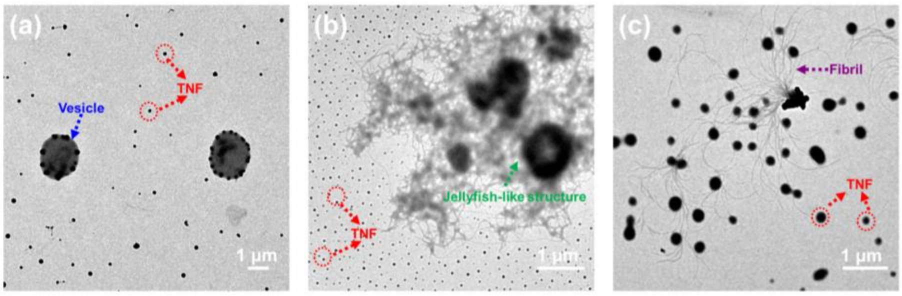

Supplementary Figure 66. Self-assembled structures with different storage times. TEM images of the assemblies obtained after TNF (*r* = 10) was added to the 2-PrOH solution of P*t*BA-*b*-PHATMA after (a) it was cooled down to r.t., (b) 15 days of storage at r.t. and (b) 120 days of storage at r.t.

Supplementary Tables

Supplementary Table 1 Molecular characterizations of $PHATMA_{14}$

M_n (kDa)	M_w/M_n	m
13.0	1.13	14

Supplementary Table 2 Molecular characterizations of P*t*BA_{102}-*b*-$PHATMA_{22}$

M_n^a (kDa)	M_w/M_n^a	M_n of P*t*BA (kDa) ^{a}	m/n	m^a	n^b
27.1	1.16	13	4.6	102	22

a: obtained from GPC; b: ^{1}H NMR spectrum.

Supplementary Table 3 Length information of the doped P*t*BA_{102}-*b*-$PHATMA_{22}$ micelles with $r = 1$ formed during the cooling process. Representative TEM images are included in Supplementary Figure 59.

T (^{o}C)	L_n (nm)	L_w (nm)	L_w/L_n	σ (nm)	$σ/L_n$
80	230	245	1.06	61	0.27
75	342	351	1.03	59	0.17
70	401	409	1.02	60	0.15
65	564	583	1.03	109	0.19
60	845	874	1.03	168	0.20
55	1977	2112	1.06	577	0.29
50	7479	7927	1.06	1755	0.23

Supplementary Table 4 Length information of the P*t*BA_{102}-*b*-$PHATMA_{22}$ micelles during the self-seeding experiment. Representative TEM images are included in Supplementary Figure 61.

T (^{o}C)	L_n (nm)	L_w (nm)	L_w/L_n	σ (nm)	$σ/L_n$
20	121	130	1.07	33	0.27
30	135	140	1.04	31	0.23
40	141	147	1.04	30	0.21
45	154	160	1.04	30	0.19
50	159	164	1.03	29	0.18
55	171	178	1.04	34	0.20
60	207	213	1.03	35	0.17
65	270	278	1.03	48	0.18
68	364	373	1.02	56	0.15
70	430	444	1.03	77	0.18
72	611	623	1.02	87	0.14

Supplementary References:

1. Huang, D. *et al.* In situ studies on the positive and negative effects of 1,8-diiodoctane on the device performance and morphology evolution of organic solar cells. *Nucl. Sci. Tech.* **32**, 57 (2021).
2. Zhao, N., Yang, C., Bian, F., Guo, D. & Ouyang, X. SGTools: a suite of tools for processing and analyzing large data sets from in situ X-ray scattering experiments. *J. Appl. Crystallogr.* **55**, 195-203 (2022).
3. Wang, Z., Sun, B., Lu, X., Wang, C. & Su, Z. Molecular orientation in individual electrospun nanofibers studied by polarized AFM-IR. *Macromolecules* **52**, 9639-9645 (2019).
4. Dazzi, A. & Prater, C.B. AFM-IR: technology and applications in nanoscale infrared spectroscopy and chemical imaging. *Chem. Rev.* **117**, 5146-5173 (2017).
5. Usov, I. & Mezzenga, R. Fiberapp: an open-source software for tracking and analyzing polymers, filaments, biomacromolecules, and fibrous objects. *Macromolecules* **48**, 1269-1280 (2015).
6. Xu, J., Ma, Y., Hu, W., Rehahn, M. & Reiter, G. Cloning polymer single crystals through self-seeding. *Nat. Mater.* **8**, 348-353 (2009).
7. Qian, J. *et al.* Self-seeding in one dimension: a route to uniform fiber-like nanostructures from block copolymers with a crystallizable core-forming block. *ACS Nano* **7**, 3754-3766 (2013).
8. Qian, J. *et al.* Self-seeding in one dimension: an approach to control the length of fiberlike polyisoprene-polyferrocenylsilane block copolymer micelles. *Angew. Chem. Int. Ed.* **50**, 1622-1625 (2011).
9. Li, X. *et al.* Monodisperse cylindrical micelles of controlled length with a liquid-crystalline perfluorinated core by 1D "self-seeding". *Angew. Chem. Int. Ed.* **55**, 11392-11396 (2016).
10. Mandelkern, L., Allou, A.L., Jr. & Gopalan, M.R. Enthalpy of fusion of linear polyethylene. *J. Phys. Chem.* **72**, 309-318 (1968).
11. Lammertink, R.G.H., Hempenius, M.A., Manners, I. & Vancso, G.J. Crystallization and melting behavior of poly(ferrocenyldimethylsilanes) obtained by anionic polymerization. *Macromolecules* **31**, 795-800 (1998).
